# Transcriptomic Responses of *Salmonella enterica* Serovars Enteritidis in Sodium Hypochlorite

**DOI:** 10.3389/fcimb.2022.853064

**Published:** 2022-04-20

**Authors:** Sheng Wang, Xingning Xiao, Mengjia Qiu, Wensi Wang, Yingping Xiao, Hua Yang, Yali Dang, Wen Wang

**Affiliations:** ^1^ State Key Laboratory for Managing Biotic and Chemical Threats to the Quality and Safety of Agro-Products, College of Food and Pharmaceutical Sciences, Ningbo University, Ningbo, China; ^2^ State Key Laboratory for Managing Biotic and Chemical Threats to the Quality and Safety of Agro-Products, MOA Laboratory of Quality & Safety Risk Assessment for Agro-Products (Hangzhou), Institute of Agro-Product Safety and Nutrition, Zhejiang Academy of Agricultural Sciences, Hangzhou, China; ^3^ School of Health Science and Engineering, University of Shanghai for Science and Technology, Shanghai, China; ^4^ College of Food Science and Engineering, Wuhan Polytechnic University, Wuhan, China

**Keywords:** cell membrane damage, membrane transport function, energy metabolism, oxidative stress response, DNA repair

## Abstract

*Salmonella enterica* serovars Enteritidis (*S*. Enteritidis) can survive extreme food processing environments including bactericidal sodium hypochlorite (NaClO) treatments generally recognized as safe. In order to reveal the molecular regulatory mechanisms underlying the phenotypes, the overall regulation of genes at the transcription level in *S*. Enteritidis after NaClO stimulation were investigated by RNA-sequencing. We identified 1399 differentially expressed genes (DEG) of *S*. Enteritidis strain CVCC 1806 following treatment in liquid culture with 100 mg/L NaClO for 20 min (915 upregulated and 484 downregulated). NaClO stress affects the transcription of genes related to a range of important biomolecular processes such as membrane damage, membrane transport function, energy metabolism, oxidative stress, DNA repair, and other important processes in *Salmonella enterica*. First, NaClO affects the structural stability of cell membranes, which induces the expression of a range of outer and inner membrane proteins. This may lead to changes in cell membrane permeability, accelerating the frequency of DNA conversion and contributing to the production of drug-resistant bacteria. In addition, the expression of exocytosis pump genes (*emrB*, *yceE*, *ydhE*, and *ydhC*) was able to expel NaClO from the cell, thereby increasing bacterial tolerance to NaClO. Secondly, downregulation of genes related to the Kdp-ATPase transporter system (*kdpABC*) and the amino acid transporter system (*aroP*, *brnQ and livF*) may to some extent reduce active transport by bacterial cells, thereby reducing their own metabolism and the entry of disinfectants. Downregulation of genes related to the tricarboxylic acid (TCA) cycle may drive bacterial cells into a viable but non-culturable (VBNC) state, resisting NaClO attack by reducing energy metabolism. In addition, significant upregulation of genes related to oxidative stress could mitigate damage caused by disinfectants by eliminating alkyl hydroperoxides, while upregulation of genes related to DNA repair could repair damage to bacterial cells caused by oxidative stress. Therefore, this study indicated that *S*. Enteritidis has genomic mechanisms to adapt to NaClO stress.

## Introduction

*Salmonella enterica* is one of the most common causes of human salmonellosis in China among the > 2600 known *S*. *enterica* serovars (Zhang et al., 2020). *S*. Enteritidis primarily causes foodborne gastroenteritis that is characterized by diarrhea, fever, headache, abdominal pain, nausea and vomiting (Daniel et al., 2015). A previous study utilizing samples from 5 pig slaughterhouses in Sardinia (Italy) had identified *Salmonella* isolates from 26/85 (30.5%) mesenteric lymph nodes, 14/85 (16.4%) colonic contents, 12/85 (14.1%) carcasses and livers from 462 samples (Piras et al., 2011). A recent study in China found that 37.5% of poultry samples were contaminated with *Salmonella* (Yang et al., 2020).

*Salmonella* can survive in a variety of adverse environments in which they have adapted to different environmental stressors ranging from nutrient starvation, acidity, osmosis and temperature fluctuations to the presence of antimicrobials (Spector et al., 2012; Bai et al., 2021). Chlorine is the antimicrobial chemical most widely used by the poultry industry due to its antimicrobial efficacy, convenience and low cost. Chilling water treatment facilities typically add between 50 and 100 mg/L of sodium hypochlorite (NaClO) to disinfect and combat microbial re-growth (Xiao et al., 2019). The antimicrobial effect of NaClO is mainly due to DNA strand breakage but it can also alter membrane permeability and inhibit transport as well as catalyze protein and nucleic acid fragmentation (Gray et al., 2013). It is believed that the mechanism of the germicidal activity of HClO or ClO^-^ is due to the inhibition of enzyme activity essential for the growth, damage to the membrane and DNA, and perhaps an injury to membrane transport capacity although it has not been fully elucidated (Fukuzaki, 2006). On the other hand, resistance to bacterial disinfectants can develop by acquiring exogenous mobile genetic elements or through inherent genetic adaptation such as antimicrobial stress-induced gene expression and mutations. These genes encode efflux pumps, membrane proteins, degradable enzymes and antimicrobial targets (Tong et al., 2021). For example, the study of Xiao et al. (2022) reported that 98.9% of *Salmonella* isolates originated from poultry showed higher NaClO tolerance. However, the transcriptome genomic mechanisms of *S*. Enteritidis to adapt to NaClO stress has not been fully elucidated.

Previous studies have used negative selection screens of mutant libraries and *in vivo* expression techniques (Raspoet et al., 2014). However, epidemiologically distinct *S*. Enteritidis strains were found to display similar gene content, which poses a major challenge to define gene function in the context of infection. Therefore, new methods are needed to analyze gene expression in enterobacteria strains (Chiok et al., 2019). Transcriptome sequencing (RNA-Seq) is a high-throughput sequencing approach to determine the sequence of all RNA transcripts in a given specimen at a given time (Daniel et al., 2015). RNA-Seq has been applied to uncover global changes in bacterial gene expression (Li et al., 2021). In particular, RNA-Seq was used to analyze the *Salmonella* Newport transcriptome after exposure to NaClO and identified differentially expressed genes (DEG) that included cellular functions such as biosynthesis, degradation, energy generation and other non-metabolic functions (Dunn et al., 2020). Wang et al. (2010) studied the transcriptomic responses of *Salmonella* typhimurium LT2 and *Salmonella enterica* PT4 under sublethal chlorine exposure and showed that 33 stress-related genes (*dnaK*, *hns*, *soxR*, etc.) and 19 virulence-related genes (*prgK*, *invI*, *invC*, etc.) in them differed in response to chlorine stress. However, the study did not perform GO analysis and KEGG analysis to annotate the key pathways. Therefore, the current study still has some limitations, and their transcriptomic mechanisms in response to *Salmonella* enterica under hypochlorite stress have not been fully elucidated.

In the current study, we examined the response of the foodborne pathogen *S*. Enteritidis CVCC 1806 to the NaClO treatment using RNA-Seq. We annotated the DEGs by BLAST analysis of the GO database and explored the biological functions of the genes by KEGG analysis. The most abundant pathways linking DEGs to phenotypic changes in each category were further analyzed. The results of this study can assist studies of stress response mechanisms and assist the application of NaClO in industrial production facilities.

## Materials and Methods

### Bacteria Preparation

*S*. Enteritidis CVCC 1806 was obtained from the China Veterinary Culture Collection Center and was stored in brain heart infusion broth (BHI, Becton Dickinson, Franklin Lakes, NJ, USA) containing 20% glycerol at -80°C until use. For use in experimental procedures, strain were incubated in 5 ml BHI at 37°C with 150 rpm for 24 h to approximately 9 log CFU/mL. Bacterial cells were collected by centrifugation (5424R high-speed frozen centrifuge, Eppendorf, Germany) at 8000 rpm for 5 min and washed three times with normal sterile saline.

### NaClO Treatment

NaClO stock solutions contained 56.8 mg/mL chlorine (Sangon Biotech, Shanghai, China) that was diluted with sterile Milli-Q water (Pall, Buckinghamshire, UK). Chlorine concentrations were 100 mg/L (pH = 11) as determined using a ChlorSense meter (Palintest, Gateshead, Tyne & Wear, UK). In bacterial suspensions, add 0.5 ml of NaClO to 0.5 ml of cell suspension for 20 min as the treatment group and treat with 0.5 ml of 0.9% NaCl as the control group, each in triplicate, and the suspension was then added to a test tube containing 100 ul of 0.1 mol/L Na_2_S_2_O_3_ to instantly burst the residual disinfectant. 0.9% NaCl can maintain the stability of intracellular osmotic pressure, while Na_2_S_2_O_3_ neutralizes the residual chlorine after the reaction to achieve the purpose of stopping the reaction (Weerasooriya et al., 2021; Xiao et al., 2022). In China, 50 to 100 mg/L NaClO without pH adjustment is commonly used in poultry chilling process and previous study has been reported that 100 mg/L NaClO treatment could promote bacteria disinfectant resistance. Therefore, 100 mg/L NaClO without pH adjustment was chosen to further investigate the transcriptomic changes to uncover the molecular regulatory mechanisms that underlie the phenotypes.

### cDNA Library Construction and Sequencing

The cDNA library construction and sequencing were performed by LC-Biotech. Hangzhou, China. RNA-Seq strand-specific libraries were prepared using the TruSeq RNA sample preparation kit from Illumina (San Diego, CA, USA) using 5 μg of total RNA. In brief, rRNA removal by RiboZero rRNA removal kit, fragmented using fragmentation buffer. cDNA synthesis, end repair, A-base addition and ligation of the Illumina-indexed adaptors were performed according to Illumina’s protocol, all of which are performed according to the manufacturer’s specifications. Screening of 200-300 bp cDNA target fragments by 2% Low Range Ultra agarose electrophoresis (BioRad, Hercules, CA, USA), followed by 15 PCR cycles of PCR amplification using Phusion DNA polymerase (New England Biolabs, Beverley, MA, USA). The amplicons were quantified using a Turner Tbs-380 fluorometer (Promega, Madison, WI, USA) and paired-end libraries were sequenced using the Illumina NovaSeq 6000 sequencing platform.

### Bioinformatic Data Analysis

Transcripts that were altered with a |fold change (FC) ≥ 2| were identified using Bioconductor edgeR (http://www.r-project.org/) and a threshold false discovery rate (FDR) of < 0.05 were considered as significant DEGs. In brief, a negative binomial distribution statistical model is built to test the original hypothesis on the data and obtain the pvalue information for gene comparisons. DEGs were subjected to enrichment analysis using Gene Ontology (GO) functions and Kyoto Encyclopedia of Genes and Genomes (KEGG) pathways Goatools (https://github.com/tanghaibao/Goatools) and Kobas (http://kobas.cbi.pku.edu.cn/home.do), respectively. DEGs were significantly enriched in GO terms and metabolic pathways when their Bonferroni-corrected *P*-value was < 0.05. Pathway-based database like KEGG helps to further analyze the biological functions of genes. Pathway enrichment analysis identified significantly enriched metabolic pathways or signal transduction pathways in DEGs comparing with the whole genome background.

### Quantitative Real-Time Reverse Transcription PCR Validation (qRT-PCR)

Representative DEGs were additionally verified using qRT-PCR using a 7300 Plus Real-Time PCR System (Thermo Fisher, Pittsburg, PA, USA). Total RNA was reverse-transcribed into cDNA using an All-in-One RT Master Mix and qPCR reactions utilized Eva Green qPCR Master Mix (Applied Biological Materials, Vancouver, Canada) with the following cycles: 95°C for 10 min followed by 40 cycles of 95°C for 15 s, 63°C for 30 s. The relative expression level of target genes was measured with the 2^-ΔΔCt^ method (Kenneth et al., 2001) and 16S rRNA was used as the reference gene. All tests were performed in triplicate using primers listed in **Table 1**.

**Table 1:** The qPCR verification of differentially expressed genes of *S*. Enteritidis CVCC 1806.

Genes	Primer sequence	Description	qPCR	RNA-seq
16S RNA	5′-AGAGTTTGATCCTGGCTCAG-3′	—	—	—
	5′-ACGGGCGGTGTGTRC-3			
*argG*	5′-GATGGCACTGCTGCACATTG-3′	argininosuccinate synthetase	-1.4↓	-1.5↓
	5′-CGTGATACTGCTCAATGGTGTCT-3			
*sucB*	5′-GAGCGTCTACTGGAAGCGAAA-3′	2-oxoglutarate dehydrogenase	-1.7↓	-1.5↓
	5′-GAGCGTCTACTGGAAGCGAAA-3			
*leuB*	5′-GCTCTATCCTGTGGCGTGAA-3′	3-isopropylmalate dehydrogenase	-2.5↓	-1.8↓
	5′-CAACTGCATGGTGGCGTTATC-3			
*dmsB*	5′-GTGGTGAACGAAGAGGTCTGT-3′	dimethylsulfoxide reductase subunit B	-1.7↓	-1.8↓
	5′-GTCATAACAGCCATCGCACTTG-3			
*cydB*	5′-TGGTGATTGGCGTGGCCTTT-3′	cytochrome d ubiquinol oxidase subunit I	2.3 ↑	1.2 ↑
	5′-CGGTGTAGTACAGACGCAGAT-3			

—, not applicable; ↓, downregulated; ↑, upregulated.

### Sequence Accession Numbers

The raw data reported in this article are available in the NCBI Sequence Read Archive at https://www.ncbi.nlm.nih.gov/bioproject/PRJNA758059 accessed on 22 September 2021 under BioProject accession number PRJNA758059.

## Results

### DEG Distributions

**Figure 1:**
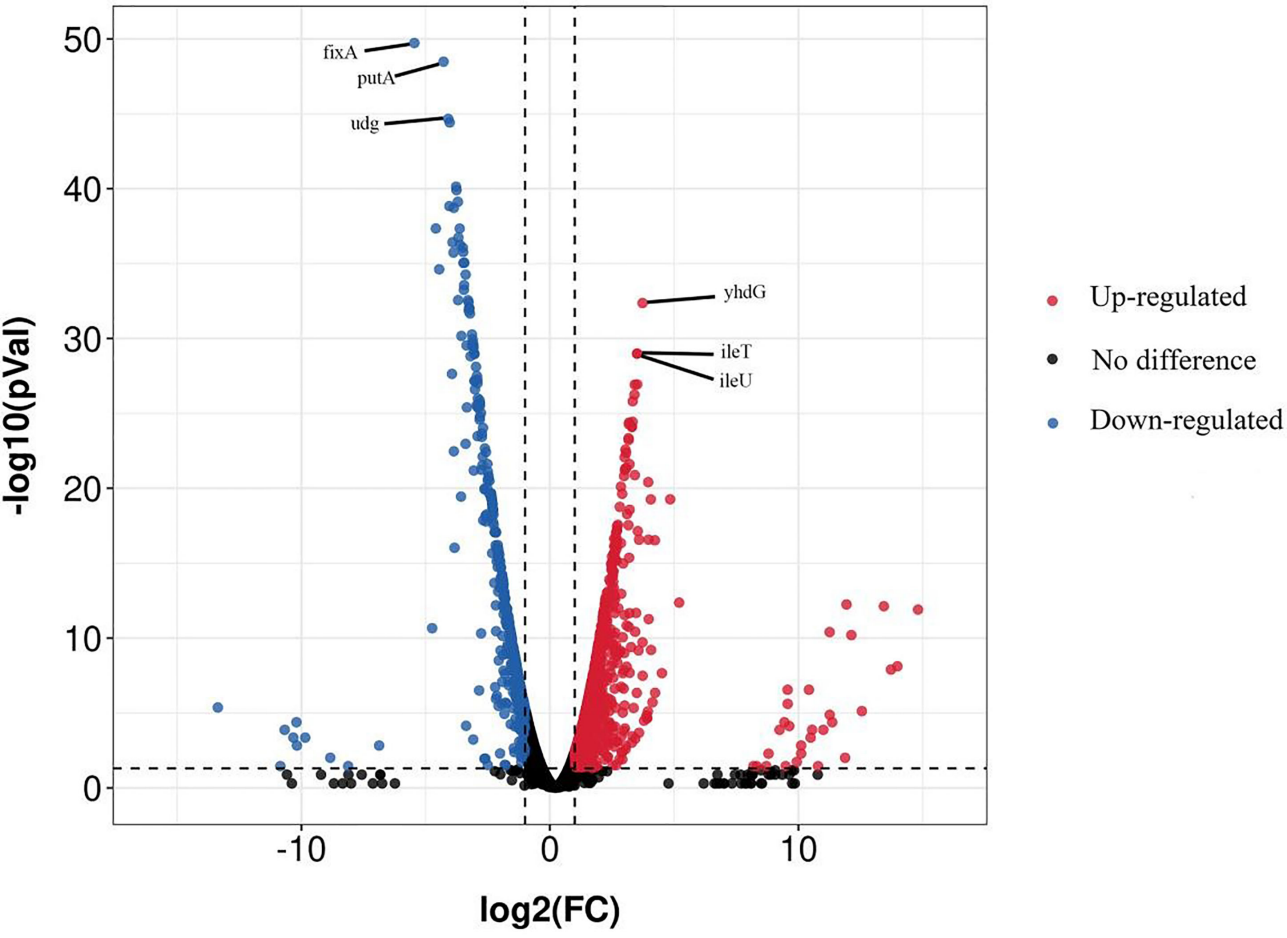
Volcano plot of *S*. Enteritidis CVCC 1806 treated with 100 mg/L NaClO. Red spots in the right part represent upregulated genes; blue spots in the left part represent downregulated genes; black spots in the middle part represent genes with insignificant changes between the stressed and unstressed.

The whole genome sequencing of our samples cultured for 20 min in the presence and absence of 100 mg/L NaClO generated 31710116 and 32912302 clean raw reads with an average length of 100 nt, respectively. A total of 4650 genes were identified using RNA-Seq and 1399 were significant DEGs. These included 915 upregulated and 484 downregulated genes and the relative levels of expression varied. As shown in **Figure 1** volcano plot diagrams revealed upregulated and downregulated DEGs of the NaClO treatment group in contrast. Among them, *yhdG*, *ileT* and *ileU* are most significantly revised upwards by 3.7 log2(FC), 3.5 log2(FC) and 3.5 log2(FC), *fixA*, *putA* and *udg* are most significantly revised downwards by 5.4 log2(FC), 4.2 log2(FC) and 4.0 log2(FC), respectively.

### GO Analyses

**Figure 2:**
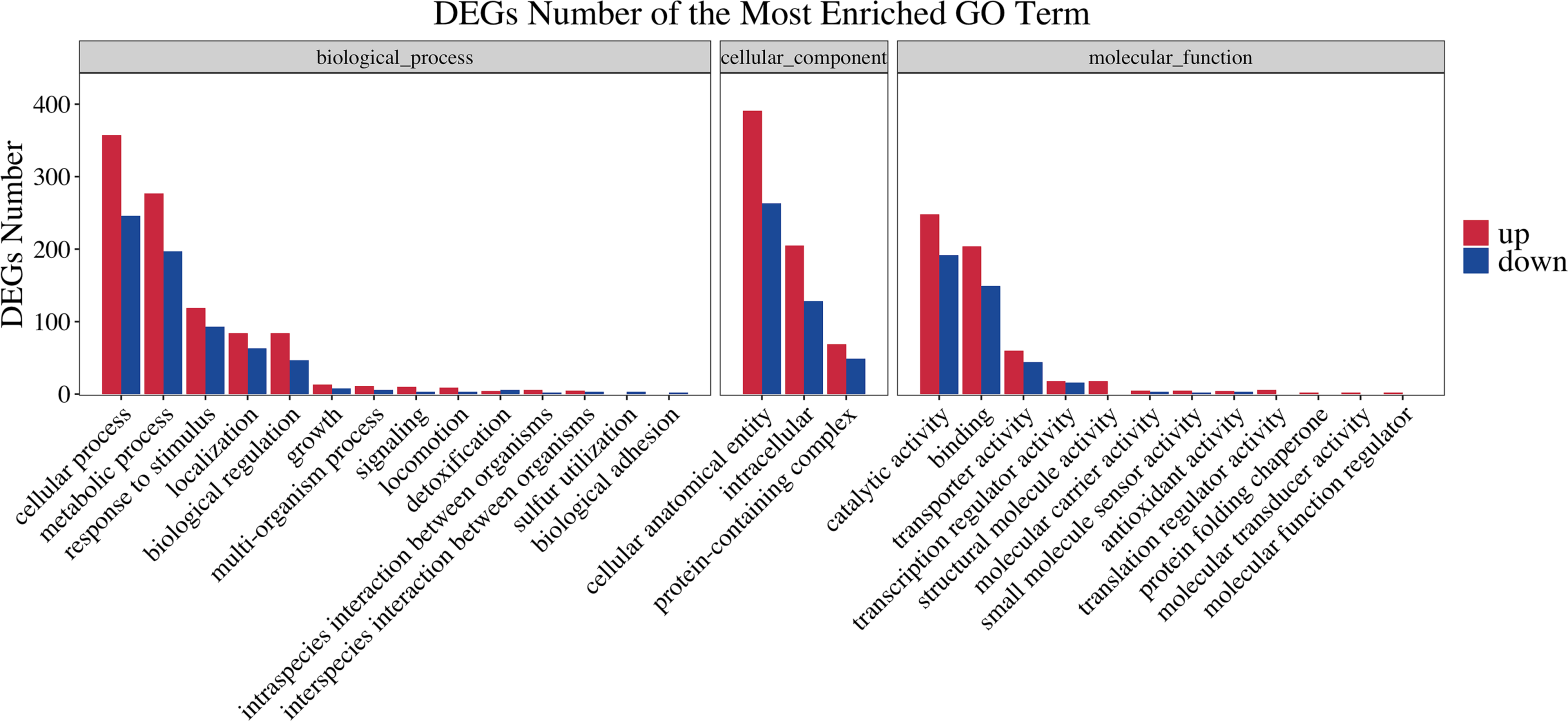
GO functional classification of differentially expressed genes between control and NaClO treated group. The x-axis denotes the subcategories and the left y-axis denotes the number of DEGs.

**Figure 3:**
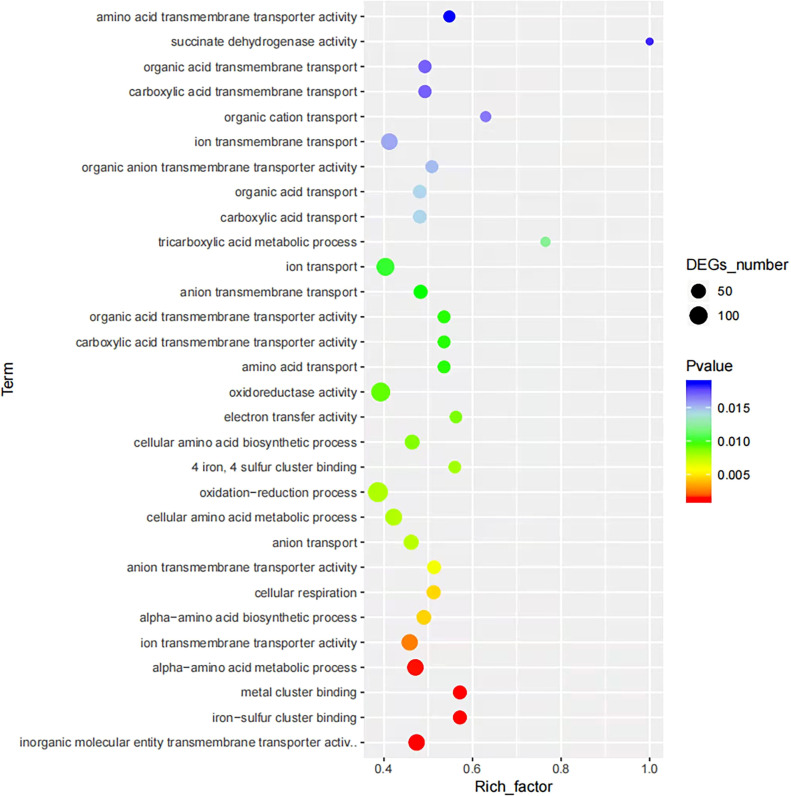
Enrichment maps of differentially expressed gene pathways between the control and NaClO treatment groups.

To obtain an overview of the changes of gene functions after NaClO treatment, the DEGs were annotated by BLAST analysis of the GO database. Within these samples we identified 363 DEGs that were annotated in biological process, 296 in cellular components and 228 in molecular function. A ranking of these 3 GO lists according to their enrichment scores indicated that the biological process DEGs were gathered at the terms ‘cellular process’, ‘metabolic process’, ‘response stimulus’, ‘localization’ and ‘biological regulation’. The cellular component terms were primarily ‘cellular anatomical entity’, ‘intracellular’, ‘protein-containing complex’ while the molecular function terms gathered at ‘catalytic activity’, ‘binding’ and ‘transporter activity’ (**Figure 2**). In the enrichment map of differentially expressed gene pathways, we found that “inorganic molecular entity transmembrane transporter activity”, “metal cluster binding” and “iron-sulfur cluster binding” were the most significantly expressed pathways (**Figure 3**).

### KEGG Analyses

**Figure 4:**
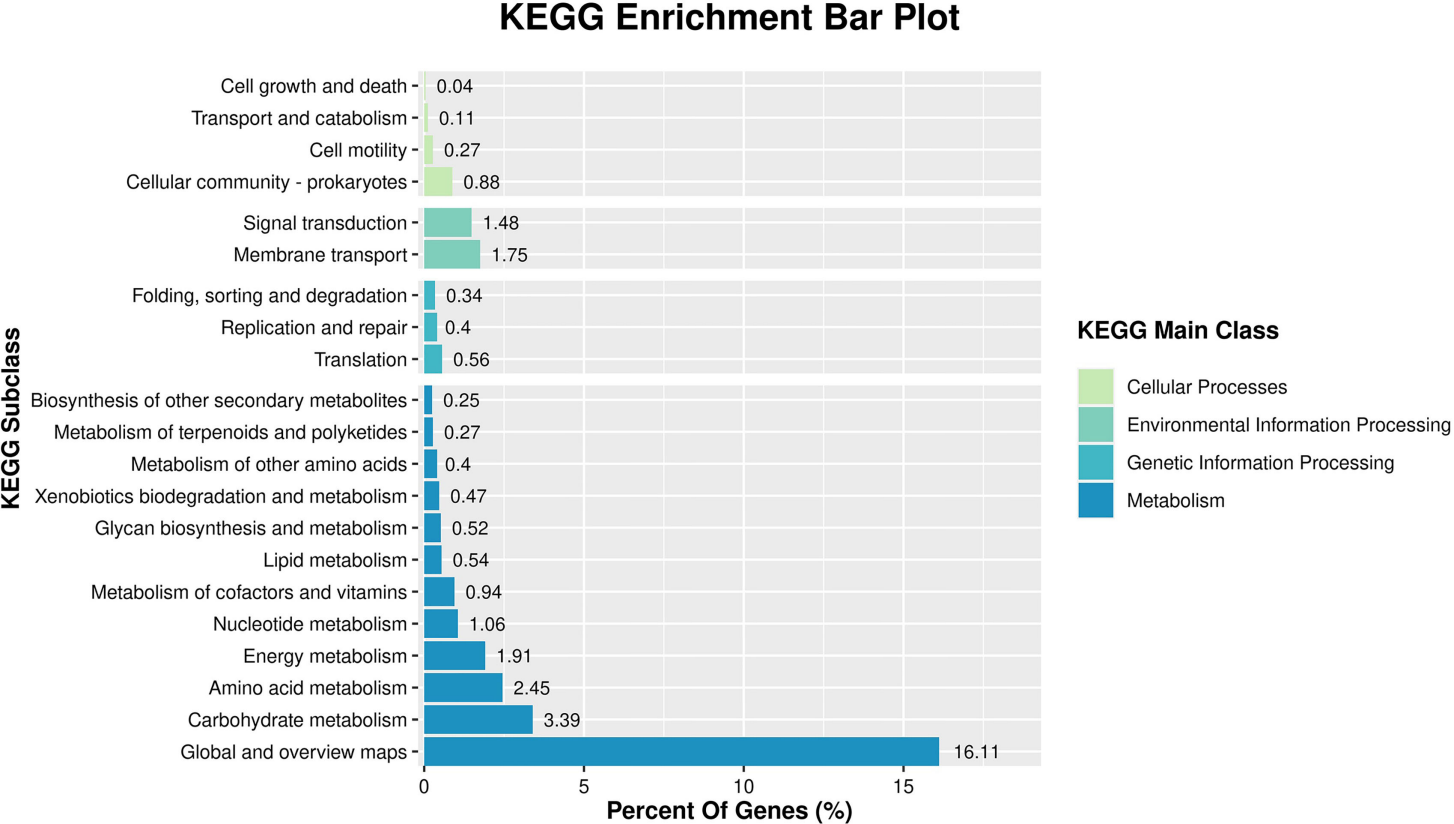
KEGG pathway classification of differentially expressed genes between control and NaClO treatment groups.

KEGG analysis was performed to explore gene biological functions and identify pathways for the DEGs. This analysis indicated that most DEGs were involved in metabolism, genetic information processing, cellular processes and environmental information processing. The metabolism category revealed groupings distributed in carbohydrate, amino acid and energy metabolism. Two enriched terms ‘membrane transport’ and ‘signal transduction’ were the primary elements for environmental information processing and the most enriched term in the cellular process category was ‘cellular community’ (**Figure 4**).

### qRT-PCR Validation

We validated the RNA-Seq results using qRT-PCR on five randomly selected DEGs. Five genes including one upregulated genes and four downregulated gene were selected for qRTPCR analysis and used to validate the RNA-seq data from preliminary experiments. In general, the qRT-PCR results were consistent with the RNA-Seq results (R^2^ = 0.93) indicating the transcriptome data were reliable (**Table 1**).

## Discussion

**Figure 5:**
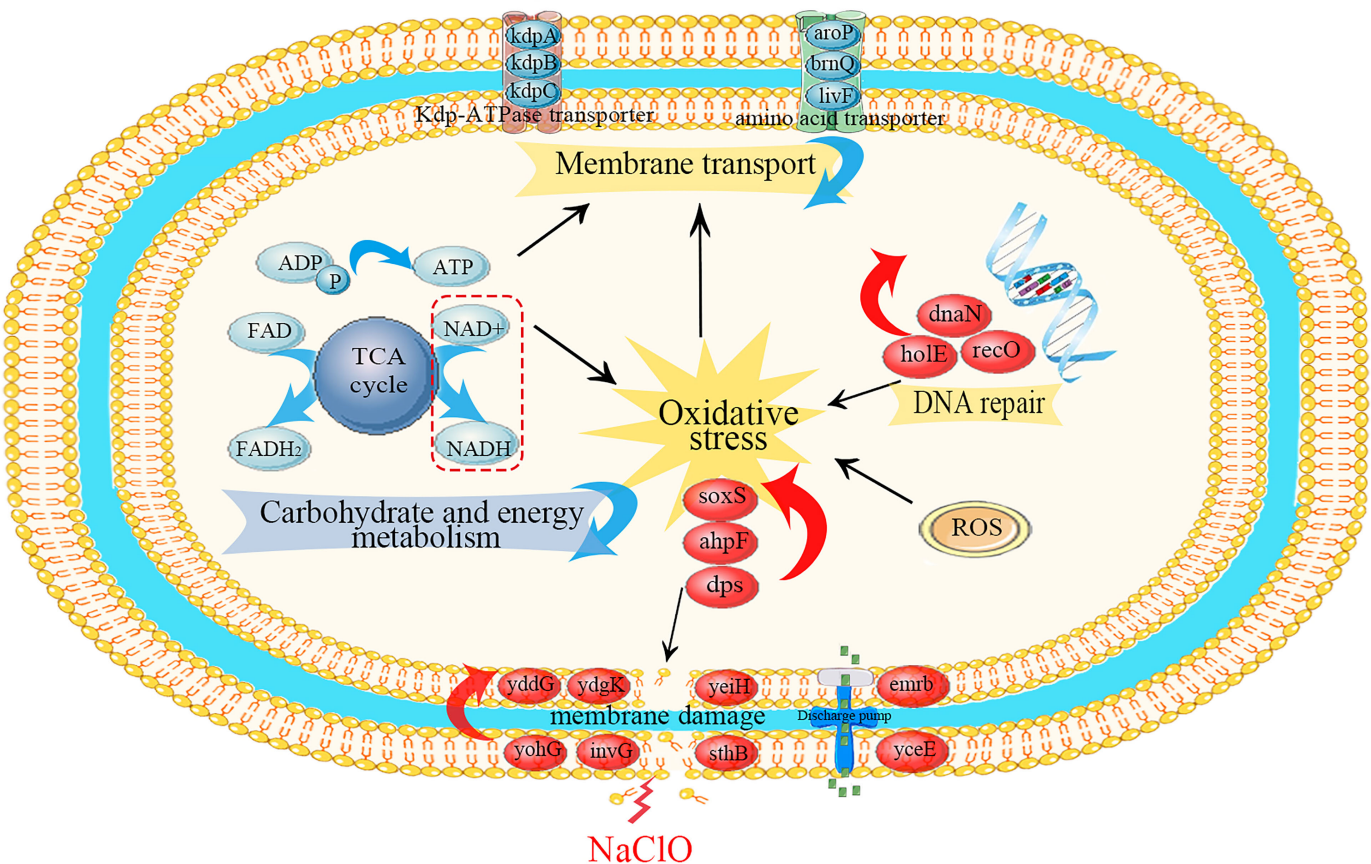
Schematic diagram of the primary metabolic regulations of *S*. Enteritidis CVCC 1806 cells under NaClO stress. Red ovals indicate upregulated differential genes, red arrows indicate up-regulation of differential genes on related pathways, blue arrows indicate downregulation of differential genes on related pathways, and black arrows indicated the activation regulation.

Our experiments revealed that gene regulation at the transcriptional level in *S*. Enteritidis CVCC 1806 was dramatically altered following a brief exposure to NaClO. The pathways most enriched were related to crucial cellular processes and we further analyzed these for each category (**Figure 5**).

### Cell Membrane Damage

In **Table 2**, DEGs encoding outer (*sthB*, *invG*, *yohG*, *apeE*, *yaiU*) and inner membranes (*ydgK*, *yddG*, *yeiH*, *yhjD*) were upregulated. This may be due to the cellular response induced by NaClO stress on *Salmonella* leading to cell membrane disruption and thus stimulating the expression of genes associated with cell membrane proteins (Zhang et al., 2021). Xiao et al. (2022) found that 100 mg/L NaClO caused damage to *Salmonella* cell membranes by flow cytometry combined with fluorescent dye technique. Rajkowski et al. (2012) also found that treatment of *Salmonella* with chlorine at a concentration of 300 ppm for 3 minutes resulted in the destruction of the outer cell membrane of *Salmonella* by transmission electron microscopy. Membrane permeability is a primary barrier to the uptake of foreign or extracellular DNA. In particular, a previous study reported that 100 mg/L NaClO exposure altered the transcription of cell membrane-related genes and especially those regulating cell membrane permeability in the bacterium *Pseudomonas* sp. (Tong et al., 2021). Additionally, following chlorine disinfection, increased membrane permeability can increase the frequency of DNA conversion (Jin et al., 2020). This releases antibiotic resistance genes into the water as free DNA, allowing chlorine-damaged conditionally pathogenic bacteria to be transferred from non-antibiotic resistant bacteria to antibiotic resistant bacteria by natural transformation during chlorination, thereby increasing the likelihood of bacterial survival (Thomas et al., 2005).

**Table 2:** Major metabolic pathways involved in differentially expressed genes.

Gene ID	Gene Name		Log2 (FC)	NR top hit description
Cell membrane damage				
STM4593	*sthB*		1.1↑	outer membrane usher protein
STM2898	*invG*		1.6↑	type III secretion system outer membrane ring protein InvG
STM2172	*yohG*		1.6↑	multidrug resistance outer membrane protein MdtQ
STM0570	*apeE*		1.2↑	outer membrane esterase
STM0373	*yaiU*		1.1↑	autotransporter outer membrane beta-barrel domain-containing protein
STM0196	*stfC*		2.0↑	fimbrial biogenesis outer membrane usher protein
STM1460	*ydgK*		1.4↑	Inner membrane protein ydgK
STM1571	*yddG*		2.4↑	inner membrane protein YddG
STM2202	*yeiH*		2.4↑	putative inner membrane protein
STM3608	*yhjD*		1.4↑	inner membrane protein YhjD
STM2815	*emrB*		1.4↑	Inner membrane component of tripartite multidrug resistance system
STM1154	*yceE*		1.2↑	multidrug efflux MFS transporter MdtG
STM1425	*ydhE*		1.4↑	multidrug efflux MATE transporter MdtK
STM1428	*ydhC*		2.6↑	Bcr/CflA family multidrug efflux MFS transporter
STM0196	*stfC*		2.0↑	fimbrial biogenesis outer membrane usher protein
STM0300	*safB*		1.2↑	putative fimbrial assembly chaparone
STM0337	*stbD*		1.2↑	fimbrial usher protein StbD
STM3619	*yhjO*		1.1↑	UDP-forming cellulose synthase catalytic subunit
STM3623	*yhjT*		1.9↑	cellulose biosynthesis protein BcsF
STM3624	*yhjU*		2.6↑	cellulose biosynthesis protein BcsG
Membrane transport function				
STM0706	*kdpC*		1.9↓	potassium-transporting ATPase subunit C
STM0705	*kdpB*		3.6↓	potassium-transporting ATPase subunit KdpB
STM0704	*kdpA*		3.7↓	potassium-transporting ATPase subunit A
STM0150	*aroP*		3.5↓	aromatic amino acid transporter AroP
STM0399	*brnQ*		1.4↓	branched-chain amino acid transporter carrier protein BrnQ
STM3560	*livF*		1.7↓	branched-chain amino acid ABC transporter ATP-binding protein LivF
Carbohydrate and energy metabolism				
STM0736	*sucA*		-1.9↓	2-oxoglutarate dehydrogenase E1 component
STM0737	*sucB*		-1.4↓	2-oxoglutarate dehydrogenase
STM0730	*gltA*		-2.5↓	type II citrate synthase
STM1238	*icdA*		-1.9↓	NADP-dependent isocitrate dehydrogenase
STM1468	*fumA*		-1.8↓	fumarate hydratase
STM0738	*sucC*		-1.4↓	ADP-forming succinate–CoA ligase subunit beta
STM0739	*sucD*		-1.2↓	succinate–CoA ligase subunit alpha
STM3290	*argG*		-1.5↓	argininosuccinate synthetase
STM0734	*sdhA*		-3.2↓	succinate dehydrogenase flavoprotein subunit
STM0733	*sdhD*		-3.0↓	succinate dehydrogenase membrane anchor subunit
STM0735	*sdhB*		-2.8↓	succinate dehydrogenase iron-sulfur protein
STM0732	*sdhC*		-3.4↓	succinate dehydrogenase cytochrome b556 subunit
STM3359	*mdh*		-2.1↓	malate dehydrogenase
STM0112	*leuB*		-1.8↓	3-isopropylmalate dehydrogenase
STM0158	*acnB*		-2.6↓	bifunctional aconitate hydratase 2/2-methylisocitrate dehydratase
STM1712	*acnA*		-1.2↓	aconitate hydratase AcnA
STM0965	*dmsB*		-1.8↓	dimethylsulfoxide reductase subunit B
Oxidative stress response				
STM0609	*ahpF*		1.5↑	Alkyl hydroperoxide reductase protein F, partial
STM4266	*soxR*		1.2↑	redox-sensitive transcriptional activator SoxR
STM2841	*ygbD*		1.5↑	NADH:flavorubredoxin reductase NorW
STM0075	*fixA*		5.4↓	putative electron transfer flavoprotein FixA
STM0936	*hcr*		2.0↑	NADH oxidoreductase Hcr
STM0735	*sdhB*		2.8↓	succinate dehydrogenase iron-sulfur protein
STM2541	*yfhF*		2.3↓	iron-sulfur cluster assembly protein IscA
STM4399	*ytfE*		2.7↓	iron-sulfur cluster repair protein YtfE
STM0831	*dps*		1.2↑	DNA starvation/stationary phase protection protein Dps
DNA repair				
STM2579	*recO*		1.3↑	DNA repair protein RecO
STM1876	*holE*		2.4↑	DNA polymerase III subunit theta
STM3837	*dnaN*		1.1↑	DNA polymerase III subunit beta
STM2414	*yfeD*		1.1↑	putative DNA-binding transcriptional regulator
STM0682	*nagC*		1.7↑	DNA-binding transcriptional regulator NagC
STM0374	*yaiV*		2.5↑	DNA-binding transcriptional regulator

↓, downregulated; ↑, upregulated.

The upregulation of exocytosis pump-related genes (*emrB*, *yceE*, *ydhE*, and *ydhC*) may also be related to the stability of cell membranes, which are capable of transporting structurally distinct molecules (including antibiotics) out of bacterial cells. Increased expression of the triple resistance system (*emrB*) genes were able to reduce disinfectant entry and express NaClO tolerance in *Burkholderia thailandensis* (Sabrin et al., 2018). This efflux lowers the intracellular antibiotic concentration, allowing bacteria to survive at higher antibiotic concentrations (Blair et al., 2014).

In addition, upregulation of genes for important components of extracellular polymersubstances (EPS) such as curly hairs (*stfC*, *safB*, *stbD*) and cellulose synthesis (*yhjO*, *yhjT*, *yhjU*) may increase cell membrane stability. NaClO solution are HClO, ClO^-^ and OH^-^ ions, and when the pH tends to be neutral, NaClO generally plays a bactericidal role in the form of HClO (Fukuzaki, 2006). On the one hand, HClO can interact with a variety of biomolecules, such as lipids, nucleic acids and membrane components, causing severe cellular damage (da Cruz Nizer et al., 2020). On the other hand, HClO increases the resistance of *Salmonella* to NaClO by causing conformational changes in membrane properties and overproduction of EPS substrates (da Cruz Nizer et al., 2020).

### Membrane Transport Function

In our study DEGs encoding the Kdp-ATPase transporter system (*kdpABC*) and the amino acid transporter system (*aroP*, *brnQ and livF*) were down-regulated in expression following NaClO treatment, which may be a protective effect of *Salmonella* against NaClO stress. Any object that enters or leaves a cell must penetrate one or more enclosing membranes (Rees et al., 2009). Channel proteins are essential for the passage of molecules (including disinfectant molecules) through the cell membrane and are essential channel components. The ATP-binding cassette (ABC) transporters are a superfamily of membrane-associated bacterial proteins that are responsible for the ATP-powered translocation of numerous types of substrates (Li et al., 2021). The *kdpABC* encodes a potassium transport channel that is required for ATP homeostasis. K^+^ is essential for many cellular functions, including maintenance of intracellular pH and transmembrane potential (Liu et al., 2020). Downregulation of the Kdp-ATPase system genes may go so far as to inhibit active microbial transport, inhibit microbial metabolism, and thus affect bacterial energy metabolism. In addition, the downregulation of genes related to amino acid metabolism is able to maintain low concentrations of these biomolecules in the cell. The main target of HClO action is the amino acid side chain, so the low yield of these molecules could reduce the damage caused by this disinfectant (da Cruz Nizer et al., 2020). Finally, due to the rate of reaction of HOCl with proteins, membrane transport proteins may be affected, affecting cellular homeostasis. Therefore, the downregulation of these proteins may be a protective strategy against HOCl damage.

### Carbohydrate and Energy Metabolism

We found that numerous the tricarboxylic acid (TCA) cycle DEGs were downregulated with NaClO exposure. These included genes encoding citrate synthase (*gltA*), aconitase (*acnA*, *acnB*), isocitrate dehydrogenase (*icdA*), α-ketoglutarate dehydrogenase complex *(sucA*, *sucB*), succinyl-CoA synthetase (*sucC*, *sucD*, *argG*), succinate dehydrogenase (*sdhABCD*), fumarase (*fumA*) and malate de-hydrogenase (*mdh, leuB*). TCA cycle produces the reductive equivalents for the electron transport chain and the carbon backbone for various amino acids, making it an important hub for efficient bacterial metabolism in changing environments (Noster et al., 2019). Citrate synthase is in a key position as the first enzyme of the TCA cycle and citrate is a component of biosynthetic intermediates and reduced purine nucleotides that are used for energy generation through phosphorylation reactions linked by electron transport (Park et al., 1994). The reaction catalyzed by IDH is a regulatory checkpoint and α-ketoglutaric acid produced by this reaction contributes to the synthesis of glutamic acid, a key amino acid precursor (Kobayashi et al., 2014a). The TCA cycle converts NAD^+^ into NADH and FAD into FADH_2_ and these components feed electrons into the oxidative phosphorylation pathway (Gel'Man et al., 1967; Li et al., 2021). Therefore, decreasing the overall yields of the TCA cycle would most likely lead to an overall decrease in cellular energy charge. Limited energy contributes to the quiescence of growth and metabolism, which may subsequently lead to the formation of the viable but non-culturable (VBNC) state (Zhu et al., 2021). Moreover, VBNC state cells will have greater resistance to chlorine (Dong et al., 2020). In addition, the dimethylsulfoxide reductase subunit B encoded by *dmsB* is able to transfer electrons, and it is significantly downregulated by downregulating 1.8 log2(FC) may everywhere lead to a decrease in the energy generated (Molinas et al., 2011). If this is a functional adaptation of this bacterium, the mechanism is currently unclear. In contrast, the overall adaptation in other metabolic pathways that we identified may be directly related to the overall decrease in cellular energy charge.

### Oxidative Stress Response

Oxidative stress and production of reactive oxygen species (ROS) is a natural consequence of aerobic metabolism (Chiang et al., 2012). Under aerobic conditions, the disinfection effect is largely caused by the damage caused by elevated ROS levels (Brynildsen et al., 2013). Our results indicated that DEGs related to oxidative stress (*ahpF*, *soxR*, *ygbD*, *hcr)* were upregulated. Generally, there are two major stress response pathways required in *Salmonella* when coping with oxidative stress; peroxide stress-response and superoxide stress-response systems (Bai et al., 2021). In living cells, elimination of alkyl hydroperoxides is particularly important since they can initiate lipid peroxidation and consequently propagate free radicals leading to DNA and membrane damage (Rocha et al., 1999). Alkyl hydroperoxide reductase encoded by *ahpF* significantly reduces the frequency of oxygen-dependent mutations caused by oxidative DNA damage. In enteric bacteria this is regulated *via soxR* that upregulates superoxide dismutase (SOD), the outer-membrane drug efflux protein TolC and DNA repair-related endonuclease IV (Kobayashi et al., 2014b). Antioxidant enzymes may be overexpressed to protect the receptor from damage caused by elevated ROS levels (Bae et al., 2011). ROS are mainly generated by the autoxidation of reduced flavoproteins that are not involved in the respiratory chain, and in our study *fixA* was significantly downregulated by 5.4 log2(FC) may also be a strategy to cope with ROS (Imlay et al., 2013). In addition, the upregulation of the NADH oxidoreductase *hcr* is another gene that responds to oxidative stress. NADH is a primary cofactor involved in detoxification and elevated NADH/NAD^+^ ratios are associated with oxidative stress (Ying, 2008). These reactions greatly increase the resistance of cells to oxidants.

On the one hand, HClO also reacts with the iron centres in microbial enzymes, leading to enzyme inactivation (Fukuzaki, 2006). On the other hand, HClO can also cause damage to DNA (Storz et al., 1999). In our study, the gene (*sdhB*, *yfhF*, *ytfE*) associated with the iron-sulfur cluster reaction was downregulated. The unscheduled transfer of electrons from oxidoreductase to oxygen produced a mixture of + ${\text{o}}_{2}^{-}$ and H_2_O_2_ (Imlay, 2019). These oxidized the solvent-exposed iron centres of the mononuclear Fe^2+^ enzyme and [4Fe-4S] dehydratase, causing iron dissociation and loss of activity (Imlay, 2019). As iron is required for the Fenton reaction, higher levels of unincorporated cellular iron may increase DNA damage (Touati et al., 1995). Therefore, downregulated iron-sulfur cluster genes may be critical for cells to cope with this damage. In addition, upregulated *dps* is a miniature ferritin that keeps free iron levels low enough to inhibit damage to DNA by sequestering loose iron from the cell (Altuvia, 1994).

### DNA Repair

DNA repair system can repair damage caused by oxidative stress (Buchmeier et al., 1995). In the current study, we identified significantly upregulated DEGs associated with DNA repair and replication, including *recO*, *holE* and *dnaN*, which were upregulated by 1.3 log2(FC), 2.4 log2(FC) and 1.1 log2(FC), respectively. Slight upregulation of nucleoside and nucleotide biosynthesis when *Salmonella* is exposed to disinfectants such as NaClO and peroxyacetic acid (PAA), which triggers DNA repair mechanisms (Dunn et al., 2020). DNA polymerase encoded by *dnaN* is able to repair damage caused by H_2_O_2_ (Burton et al., 2007). Therefore, it is critically important for a cell to have the capacity to properly respond to and repair DNA damage as it occur (Minten et al., 2019). Interestingly, we also identified DEGs involved in transcriptional regulation that are key elements in environmental adaptation (Li et al., 2021) including *yaiV*, *nagC* and *yfeD*. Under NaClO stress, ribosome synthesis is enhanced, stimulating DNA repair functions and possibly promoting translation to some extent, which may contribute to increased resistance of *Salmonella* to NaClO.

## Conclusions

Transcriptional upregulation of genes associated with key cellular processes such as membrane damage oxidative stress and DNA repair was observed under NaClO stress. On the one hand, altered cell membrane permeability and increased pore space accelerated the frequency of DNA transfer from non-drug-resistant to drug-resistant bacteria through natural transformation. On the other hand, upregulation of efflux pump genes enables the exclusion of disinfectants from the bacterial cell body. *Salmonella* can reduce DNA damage by eliminating alkyl hydroperoxides, while upregulation of DNA repair-related genes can repair DNA damage induced by oxidative stress, thereby reducing damage to *Salmonella* by NaClO. However, we also identified downregulation of genes related to membrane transport function and energy metabolism, which may reduce active transport in bacteria and force them into a VBNC state to resist NaClO attack by reducing their own metabolism. Therefore, this study suggests that *Salmonella enterica* has a genomic mechanism to adapt to NaClO stress, which may provide a reference for the application of NaClO in industrial production facilities.

## Data Availability Statement

The datasets presented in this study can be found in online repositories. The names of the repository/repositories and accession number(s) can be found in the article/supplementary material.

## Author Contributions

YD and WenW: writing-review and editing. SW and XX investigation and writing-original draft preparation. MQ and WensiW data curation. YX and HY: resources. All authors contributed to manuscript revision, read, and approved the submitted version.

## Funding

This research was supported by State Key Laboratory for Managing Biotic and Chemical Threats to the Quality and Safety of Agro-products (2010DS700124-ZZ2002), Walmart Foundation (UA2020-152, UA2021-247) and Ministry of Agriculture and Rural Affairs (14215033).

## Conflict of Interest

The authors declare that the research was conducted in the absence of any commercial or financial relationships that could be construed as a potential conflict of interest.

## Publisher’s Note

All claims expressed in this article are solely those of the authors and do not necessarily represent those of their affiliated organizations, or those of the publisher, the editors and the reviewers. Any product that may be evaluated in this article, or claim that may be made by its manufacturer, is not guaranteed or endorsed by the publisher.
